# Exploring the lived experiences of individuals with Parkinson’s disease and their relatives: insights into care provision experiences, disease management support, self-management strategies, and future needs in Germany (qualitative study)

**DOI:** 10.1186/s12883-024-03696-y

**Published:** 2024-06-18

**Authors:** Theresia Krieger, Leonie Jozwiak, Georg Ebersbach, Thorsten Suess, Björn Falkenburger, Tim Feige, Carsten Eggers, Tobias Warnecke, Winfried Scholl, Christian Schmidt-Heisch, Ann-Kristin Folkerts, Elke Kalbe, Ümran Sema Seven

**Affiliations:** 1 Medical Psychology | Neuropsychology and Gender Studies, Centre for Neuropsychological Diagnostics and Intervention (CeNDI), Faculty of Medicine, University Hospital Cologne, University of Cologne, Cologne, Germany; 2 Movement Disorder Clinic, Kliniken Beelitz, Beelitz-Heilstätten, Germany; 3 Department of Neurology, University of Technology Dresden, Dresden, Germany; 4 Knappschaftskrankenhaus Bottrop, Department of Neurology, Bottrop, Germany; 5 Department of Neurology, University Hospital Munster, Munster, Germany; 6 Young Parkinson Association, Schneckenheim, Germany; 7 Parkinson Pate Organization, Hamburg, Germany

**Keywords:** Parkinson’s disease, Qualitative research, Relatives, Lived experience, Experiences of illness, Self-management, Informational needs, Management support needs

## Abstract

**Background:**

Parkinson’s disease (PD) significantly impacts the health-related quality of life of affected individuals and their relatives. In order to support the affected individuals and their families in coping with PD, it is essential to offer comprehensive information about their experiences. A comprehensive understanding of their lived experiences with the disease, the healthcare system, applied self-management strategies and their needs is considered crucial for developing a PD support program. Therefore, we aimed to explore the lived experiences and support needs of individuals with PD and their relatives in Germany.

**Methods:**

This non-interventional, qualitative study conducted an explorative status quo and needs assessment. It generated knowledge through semi-structured focus groups and interviews with individuals with PD at various disease stages and their relatives. The interviews were digitally recorded, transcribed verbatim, and analysed using content analysis.

**Results:**

Fifty-two individuals with PD and 29 relatives participated in eight focus groups and 13 paired and 13 individual interviews. Four themes with corresponding subthemes emerged: (1) experiences, revealing individuals’ experiences around their diagnosis and with disease-specific care provision; (2) management support offers, clarifying who provides support and the type of support offered; (3) self-management, including comprehensibility, meaningfulness and manageability; and (4) future needs, differentiating between deficits and needs. Most participants expressed a sense of abandonment when obtaining self-management strategies and mastering their lives with PD, often referred to as ‘life 2.0’. They identified the lack of structured and adequate provision of information, system orientation and social awareness.

**Conclusions:**

In Germany, there is an urgent need for a comprehensive PD care program that addresses the needs of individuals with PD and their relatives from the start of their care trajectory. It could assist individuals in gaining a comprehensive understanding of the disease, obtaining self-management strategies, building a support network, and becoming experts in self-managing their disease. Moreover, it may positively influence their care trajectory and reduce burdens, such as overburdening, fear of progression, and health anxiety.

**Trial Registration:**

German Clinical Studies Register (https://www.drks.de/DRKS00030090, No. DRKS00030090, Date of registration: 15.12.2022).

**Supplementary Information:**

The online version contains supplementary material available at 10.1186/s12883-024-03696-y.

## Background

### Parkinson’s disease and its disease-related burden on affected individuals and their relatives

Parkinson’s disease (PD) is a progressive neurodegenerative disorder with a rapidly increasing prevalence that affects the lives of those affected at various levels [1]. In Germany, around 400,000 individuals are affected by PD, and the impact of PD will increase further due to the ageing population in Western societies [2].

PD’s complex nature is associated with considerable limitations in the health-related quality of life of people with PD (PwPD) and their relatives [3]. The diversity of motor symptoms (e.g. bradykinesia, rigidity, tremor and postural instability) and non-motor symptoms (e.g. cognitive dysfunction and depression) constitutes a severe burden not only for PwPD but also for their relatives, typically the spouse or a child of the person with PD [4, 5]. Clinicians have tended to frequently focus on the key motor symptoms and neglect other symptoms (e.g. depression) or needs (e.g. psychosocial issues) [6, 7]. However, non-motor symptoms strongly determine the quality of life of PwPD [8]. Moreover, ‘reducing’ PD to a merely biomedical issue is problematic since it potentially compromises the well-being of both PwPD and their relatives [9].

PD is also considered to impose a significant burden on the relatives of PwPD, including fear of disease progression [10]. Spouses or other family members can suddenly find themselves in the position of becoming an ‘informal’ caregiver, generally without financial compensation for their effort [11, 12]. Due to insufficient disease-specific social support or inadequate care provision, informal caregivers may experience constraints on their relationship and everyday life, concerns about the future and lack of employment of the PwPD, which may adversely affect their emotional and physical well-being (e.g. anxiety or depression) [10, 11, 13].

### Care and support needs

Few qualitative studies have explored the lived experiences and support needs of PwPD and their relative caregivers, especially in early-stage PD [14]. Furthermore, there is a paucity of research examining the potential for implementing an advanced care approach for this group [15]. PD requires the care of a multidisciplinary team of healthcare professionals and individualised medication [16]. Intensive interdisciplinary cooperation between healthcare professionals and a single point of contact or a helpline providing individual support is considered highly beneficial for PwPD and their families [17]. The strong involvement of PwPD in therapy decisions correlates significantly with their satisfaction regarding the consultation and distress relief [18]. Above all, a self-management approach plays a significant role in empowering PwPD and their caregivers to overcome problems, make decisions, activate resources, build physician-patient relationships and implement measures [19].

### Care and support approaches in Germany

Germany currently lacks a patient-centred, nationally coordinated and well-established holistic approach to care that focuses on both supporting PwPD and their families [20]. A lack of effective communication between healthcare professionals and people with Parkinson’s disease (PwPD) and their relatives is a significant issue. This communication gap can result in unintended changes to treatment, which may lead to suboptimal therapeutic outcomes and hospital admissions [21].

In certain German regions PD networks exist. Their objective is to enhance the quality of care for PwPD, reduce the incidence of unnecessary hospital admissions and associated costs, and facilitate the sharing of data and resources among different healthcare providers operating across various sectors [22]. However, most PD networks rely heavily on local initiatives, where consultation is primarily provided by resident neurologists and, in many cases, in specialized PD clinics [20]. Moreover, recently integrated concepts have been introduced, which have been demonstrated to enhance the quality of life and satisfaction of PwPD with regard to healthcare services [20, 21]. Meanwhile, a status quo analysis of current care practice was conducted that explored extensive routine data for Saxony in Germany, indicating a need for innovative care concepts [23].

In order to fill the gap in holistic care, a nationwide systematic PD support programme should be implemented by providing needs-based training with a focus on self-management. It should be integrated into a ‘Parkinson’s school’ for PwPD and their families [24]. To establish this school at a the federal level and ensure coverage by statutory health insurance, a number of mandatory steps must be undertaken, including development, practical adaptation, and evaluation [25]. Given the dearth of studies investigating the lived experiences of German PwPD and their relatives with the current support system and their desires regarding a disease-related school, the initial step was to conduct a systematic status quo and needs assessment within the ‘WissensPARK’ (‘KnowledgePARK’) project. This step was funded by the German Parkinson Association (Deutsche Parkinson Vereinigung; 08/2022–08/2023). WissensPARK´s aim was to explore the lived experiences of PwPD and their relatives with the diagnosis and subsequent consultation and care, as well as their ‘preferences and needs’ regarding the content and didactics of a potential self-management support program.

This article specifically reports on the first part of the larger WissensPARK study, which comprehensively assesses the current status quo and identifies the support needs of the target population. The findings regarding the desired content and didactics for the support program (school) will be published elsewhere.

### Objective

We aimed to explore the lived experiences of German PwPD and their relatives with PD, PD-related care provision, perceived disease management support, and self-management strategies, as well as their future support needs.

## Methods

### Design

WissensPARK was a non-interventional, explorative, mixed-method qualitative study [26]. Qualitative research tends to be small in order to support the depth of case-oriented analysis and to find information-rich material [26]. This approach provides a multifaceted and comprehensive understanding of the research topic [26, 27]. New knowledge was generated through semi-structured individual (II), paired (PI), and focus group (FGI) interviews. A flexible data collection approach was necessary to permit all potentially interested individuals to participate by acknowledging their PD severities and availabilities in a given time slot.

### Setting and sampling

This study used purposeful sampling [28]. Its data were collected nationwide, and recruitment efforts were facilitated through collaborations with clinics, institutes and stakeholders from the German Parkinson Association and affiliated self-help groups, who assisted in identifying and reaching out to suitable candidates (see the Acknowledgements).

In qualitative research, the concept of sample adequacy concerns the suitability of the composition and size of the sample [29]. In selecting participants, a number of parameters were taken into account, including the scope of the study, the nature of the topic (in terms of complexity and accessibility), and the quality of the data [26]. We did not aim for saturation, but our data collection strategy was focused on gaining comprehensive insights into the diverse needs of PwPD at different stages of PD, characterised by the Hoehn and Yahr stage classification [30]. In order to understand the specific needs of newly diagnosed PwPD and their relatives, we defined one special group (‘de novo’), which included PwPD who had received their diagnosis within the last two years. Our recruitment strategy was flexible to the circumstances at the respective locations and cooperation partners (e.g. consent or availability of PwPD and their relatives). Further collaboration partners, such as experts with their own experiences group ‘Parkinson Paten’ (PwPD that act as PD mentors), were also included in this study.

### Participants

The study participants were PwPD and their relatives; however, they were not required to participate in pairs. The inclusion criteria were PwPD at all Hoehn and Yahr stages, including those with new diagnoses [30], of both sexes who were aged ≥ 18 years (no upper age limit) and cognitively able to participate (assessed by the cooperating partners and researchers). We also included relatives of PwPD at all Hoehn and Yahr stages [30], regardless of whether or not they were informal caregivers. All participants had to be native German speakers or have an excellent command of it and unrestricted or sufficiently corrected vision and hearing abilities. The PwPD’s Hoehn and Yahr stage [30] was assessed by the cooperating partners or within the medical documentation of the treating neurologist before data collection.

### Data collection

The participants were asked to complete a sociodemographic information form that asked for data such as education, employment status, and living situation.

It was initially planned to collect qualitative data face-to-face in various healthcare settings specialising in PD, such as university hospitals or rehabilitation clinics. However, due to geographic distance and the PD-associated burden, three FGIs had to be conducted online via the ‘Zoom’ online meeting platform. Some participants interviewed face-to-face were inpatients at cooperating clinics, while others travelled to the respective data collection locations. Moreover, participation in an FGI was not appropriate for all participants due to their physical circumstances or availability, so they were offered a PI or II.

Between January and April 2023, data were collected by three researchers with backgrounds in psychology, nursing and public health (ÜSS, LJ and TK). Data collection was guided by the same semi-structured interview guidelines. Our instrument was based on a previously co-developed and piloted guideline used by one of the cooperating partners [31]. Our research team adapted it to our specific research needs and piloted it before data collection (Annex 1).

Interviews with PwPD and relatives were conducted separately. Whenever possible, both groups were subdivided into four groups based on the Hoehn and Yahr stage (de novo, H&Y 1, H&Y 2–3 and H&Y 4–5) [30], ensuring that PwPD were grouped with those at the same stage during data collection, and relatives were grouped with those whose affected relatives were currently at the same stage.

Two team members conducted the FGIs, while a single team member conducted the PIs or IIs. All interviews were audio recorded, and notes were taken. During face-to-face FGIs, important issues were visualised on flipcharts parallel to the data collection and used for clarification or periodisation. The photo-documented flipcharts assisted in the analysis process.

### Data analysis

This study followed the Consolidated Criteria for Reporting on Qualitative Studies (COREQ) guidelines [32]. The qualitative content analysis was conducted between April and July 2023 [33]. First, all audio files were transcribed verbatim by an external transcription bureau, considering the standards of social research [34]. Anonymisation was achieved by assigning each participant a pseudonym in the form of an ID number, which provided information only about the study site and the Hoehn and Yahr stage [30] of the PwPD or, respectively, the participating relative. Second, transcripts were analysed using MAXQDA (version 22), with three researchers (TK, LJ and ÜSS) participating in the coding process. Two researchers independently coded each transcript using a deductive-inductive content analysis [33]. The initial coding tree was created deductively in alignment with the interview guidelines. Third, based on emerging themes in the interview material, additional themes and subthemes were incorporated inductively, grouped and condensed. Illustrative quotes were identified and highlighted. In order to gain a deeper understanding of the meaning, perceived differences among the coders were discussed. The discussions between the coders and the entire research team continued until a consensus was achieved and a final coding tree was agreed upon. The quotes integrated into this article were translated from German to English by a fluent English-speaking research team member (TK).

## Results

### Sociodemographic description

Eighty-one individuals participated in WissensPARK (Table 1). The participants included inpatients at a PD clinic, PwPD living at home who travelled to the study sites to participate, members of a Young Parkinson’s group (‘JuPa-Group’), and a group of experts with their own lived experiences (‘Parkinson Paten’ [Parkinson mentors]) and their relatives. There were no dropouts; all participants completed the interviews.

**Table 1:** The participants’ basic characteristics

		PwPD			Relatives		
		Total (*N*)	Female (*n*)	Male (*n*)	Total (*N*)	Female (*n*)	Male (*n*)
		52 (100%)	22 (42.3%)	30 (57.7%)	29 (100%)	22 (75.9%)	7 (24.1%)
Age (years)	30–40	–	–	–	1 (3.7%)	–	1 (16.7%)
	41–50	7 (13.5%)	1 (4.5%)	6 (20.0%)	4 (14.8%)	4 (19.0%)	–
	51–60	17 (32.7%)	10 (45.5%)	7 (23.3%)	9 (33.3%)	8 (38.1%)	1 (16.7%)
	61–70	18 (34.6%)	7 (31.8%)	11 (36.7%)	9 (33.3%)	6 (28.6%)	3 (50.0%)
	> 70	10 (19.2%)	4 (18.2%)	6 (20.0%)	4 (14.8%)	3 (14.3%)	1 (16.7%)
Age (Min–Max)		41–77	49–77	41–75	39–76	45–75	39–76
Mean age (SD)		61.06 (9.26)	61.95 (7.76)	60.40 (10.29)	60.59 (9.64)	60.05 (8.91)	62.50 (12.63)
Education level	No formal education	1 (1.9%)	1 (4.5%)	–	–	–	–
	Primary school	1 (1.9%)	1 (4.5%)	–	2 (6.9%)	2 (9.1%)	–
	Secondary school	7 (13.5%)	2 (9.1%)	5 (16.7%)	3 (10.3%)	1 (4.5%)	2 (28.6%)
	Secondary high school	21 (40.4%)	12 (54.5%)	9 (30.0%)	11 (37.9%)	7 (31.8%)	4 (57.1%)
	Undergraduate degree	22 (42.3%)	6 (27.3%)	16 (53.3%)	13 (44.8%)	12 (54.5%)	1 (14.3%)
Vocational qualification	No vocational qualification	2 (3.8%)	1 (4.5%)	1 (3.3%)	1 (3.4%)	1 (4.5%)	–
	Vocational qualification	27 (51.9%)	14 (66.7%)	13 (43.3%)	16 (55.2%)	10 (45.5%)	6 (85.7%)
	Applied Sciences qualification	13 (25.0%)	4 (19.0%)	9 (30.0%)	7 (24.1%)	6 (27.3%)	1 (14.3%)
	University qualification	8 (15.4%)	2 (9.5%)	6 (20.0%)	5 (17.2%)	5 (22.7%)	–
	Postgraduate degree	1 (1.9%)	–	1 (3.3%)	–	–	–
Hoehn and Yahr stage	De novo* < 50 years	1 (1.9%)	1 (4.5%)	–	2 (8.0%)	–	2 (28.6%)
	De novo > 50 years	6 (11.5%)	3 (13.6%)	3 (10.0%)	7 (28.0%)	4 (22.2%)	3 (42.9%)
	Stage 1	13 (25.0%)	4 (18.2%)	9 (30.0%)	3 (12.0%)	3 (16.7%)	–
	Stage 2–3	27 (51.9%)	13 (59.1%)	14 (46.7%)	11 (44.0%)	10 (55.6%)	1 (14.3%)
	Stage 4–5	5 (9.6%)	1 (4.5%)	4 (13.3%)	2 (8.0%)	1 (5.6%)	1 (14.3%)

^*^De novo refers to PwPD who received their PD diagnoses within the last 24 months. Key: SD, standard deviation

### Interview data

New knowledge was generated from 34 qualitative data collection sessions: 8 FGIs, 13 PIs and 13 IIs. Of these, 31 were conducted face-to-face in collaboration with cooperating clinics at various locations across Germany (see the Acknowledgements); three FGIs were conducted online with participants distributed throughout Germany.

Altogether, 2836 min of audio material were recorded, with interview durations ranging from 32 to 119 min (mean = 83 min). The findings presented in this article are based on the first part of the interview guideline (the status-quo assessment, Annex 1), which constitutes approximately one-third of the audio material; the other parts focus on the support needs, emphasising information transmission (e.g. content or conditions), and will be published elsewhere.

### Outcomes

Four main themes emerged from the interviews with the PwPD and their relatives: (1) experiences, (2) PD-management support offers, (3) self-management strategies and (4) future necessities. The outcomes will be discussed sequentially, first for the PwPDs and then for the relatives.

Table 2 summarises the four main themes and their corresponding subthemes from the perspectives of the PwPD and their relatives. The quotes presented below belong to two groups: PwPD or the relatives of PwPD (labelled as ‘R’).

**Table 2:** Themes and subthemes that emerged from the lived experiences of PwPD and their relatives

Theme 1		Theme 2		Theme 3			Theme 4	
Experiences		Management support offers		Self-management			Future necessities	
**Subtheme 1**	**Subtheme 2**	**Subtheme 1**	**Subtheme 2**	**Subtheme 1**	**Subtheme 2**	**Subtheme 3**	**Subtheme 1**	**Subtheme 2**
Experiences around the diagnosis	Experiences with care provision	Supporters	Type of support	Comprehensibility	Meaningfulness	Manageability	Deficits	Needs
**Perspectives of PwPD**								
• Diagnosis transmission • Symptoms • Challenges • Reactions • Initial questions	• Involved professionals • Treatment offers • Care provision setting • Health insurance	• Specialised clinics • Parkinson’s associations • Contact person	• Individual training • Informational assembly	• Self-assessment • Conveying information • Information channels	• Acceptance • Repression • Positive mind set • ‘Enjoying today’	• Shaping ‘life 2.0’ • Network building • Family support • Household support • Relationship	• Structured and adequate information provision • Adequate time • Capabilities • System orientation • Social perceptions	• Holistic and comprehensive care concept • Structured training programme • Networks and peer support • Psychological support • Relatives support
**Perspectives of relatives of PwPD**								
• Diagnosis transmission • Challenges • Reactions and emotions • Initial questions	• Involved professionals • Treatment offers • Psychological support	• No support service • Specialised clinics • Parkinson’s associations	• Informational assembly	• Self-assessment • Conveying information • Information channels	• Acceptance • Positive mindset • Self-initiative	• Shaping ‘life 2.0’ • Communication with the children • Network building • Physical activity	• Information • Understanding treatment • System orientation • Support for relatives • Social perceptions	• Holistic and comprehensive care concept • Networks and peer support • Psychological support

## Theme 1 - experiences

### Subtheme 1

#### The lived experiences of PwPD around their diagnosis

The lived experiences of PwPD when being informed of their diagnosis ranged from ‘very good’ to ‘very unsatisfactory’. Among those who were unsatisfied, a clear pattern emerged of disappointment with how their physician delivered their diagnosis. The PwPD complained about receiving only a very brief explanation from their physician. One participant reported receiving only a phone call with no additional information other than the diagnosis, while another reported that they were first informed of their diagnosis through a casual remark made by a nurse.

For many participants, the process of identifying PD had been lengthy and debilitating, with a long period between the initial perception of symptoms and the final diagnosis. Both PwPD and their relatives reported fatiguing, exhausting feelings of uncertainty.

The described symptoms at the time of diagnosis were very diverse, with their intensity increasing as the disease progressed. They ranged from tremors and micrographia to limb pain, excessive movements, insomnia, disturbing dreams, depressive periods, swallowing difficulties, loss of smell, gait instability, stumbling or freezing of gait.

However, reaching the point of obtaining a diagnosis was perceived as challenging. Many PwPD felt that their initial symptoms (e.g. loss of smell or taste and feelings of numbness in the arms or legs) were not attributed to PD. In the search for a cause, they had to undergo a strenuous journey involving several screening procedures with medical professionals. The health of a few PwPD was even damaged due to incorrect diagnosis and, consequently, incorrect medications. For PwPD whose diagnosis was reached more quickly, the process was often driven by their intuition or premonition or a premonition from family members or friends.

Regarding their initial emotional reactions, the answers of PwPD were very heterogeneous, ranging from shock to sadness, anger, or fear. Some were overwhelmed with the information, while others immediately took the initiative. Some denied the diagnosis at first, while others felt a certain vindication about finally having a diagnosis that explained their symptoms. Some PwPD withdrew from social life. Initially, the emerging questions mainly focussed on the nature of PD, future prospects and treatment options, and independence and work life, all corresponding to a constant fear of disease progression.

### Relatives’ experiences around the diagnosis

The diagnosis was typically delivered by a neurologist. Relatives often described a similarly lengthy and debilitating process that they had to endure between the initial symptoms and the final diagnosis, which was perceived as challenging. More frequently than in the PwPD, relatives stated that they had a clear premonition about the nature of the symptoms observed in their affected relative. Even in cases where participants could not specifically connect the symptoms to PD, they simply *‘noticed that something was wrong*’ (R 2, H&Y 1). This realisation often occurred long before any physicians were involved.

Once a final diagnosis was reached, relatives often noted that they were not adequately informed, and they felt alone in dealing with the shock and fear. Some complained about not being believed about the severity of the changes they had noticed in their affected relative.

When asked about their initial emotional reactions, the relatives’ answers were just as diverse as those of the PwPD. Many described feelings of relief about finally having diagnostic certainty. Other reactions ranged from shock, helplessness, distress and grief to fear or denial.

Questions arising after receiving the diagnosis focused on general information about PD, causes and possible symptoms, treatment options, disease progression, independence, employment, available resources and entitlements. At this time, many thought about how to cope emotionally with the expected changes in their relationship and daily lives.

### Subtheme 2

#### PwPDs’ experiences with care provision

Disease-specific medical care was primarily provided by neurologists, with general practitioners occasionally involved. Finding a ‘*suitable and competent neurologist*’ was described as challenging (PwPD 41, H&Y stage 2–3). PwPD expressed frustration about feeling lost in the ‘maze’ or ‘jungle’ of different medical professionals (e.g. general physician, neurologist or physiotherapist). In contrast, those who had a stayover in a PD-specific rehabilitation clinic generally felt well-supported. Satisfaction with therapies such as physiotherapy or occupational therapy was generally good, although some PwPD felt the therapeutic effect was limited.

Medication treatment was almost always offered as the first therapeutic step. However, many PwPD expressed that the information about side effects was scarce, and especially those who experienced impulse control disorders due to their medication saw a great need for better clarification from physicians. Since medication is perceived as an ‘*abiding theme*’ (PwPD 38, de novo), a more holistic approach is universally desired by the PwPD. Deep brain stimulation was a particularly sensitive topic. Many participants expressed fear of such an intrusive operation, while others saw it as a kind of ‘last resort’ and refused it because it would mean that all measures had been taken, and there was nothing else they could hope for to ease their symptoms.

Significant differences became evident regarding how well or quickly participants gained orientation with the possible care and therapy options. This process seems to be strongly influenced by the dedication and willingness of the respective neurologist to invest time in informing the PwPD. Since everyone in Germany has health insurance, some PwPD highlighted that they had hoped for more comprehensive support, especially regarding support offers (e.g. system orientation or finding a suitable neurologist).

#### Relatives’ experiences with care provision

Family members of PwPD reported that despite the early manifestation of symptoms in their affected relatives, the diagnosis was often delayed. Criticism was directed at physicians for sometimes recognising PD belatedly. Typically, the diagnosis was communicated by a neurologist. Family members often perceived insufficient information and found themselves in shock after the diagnosis. Physician-patient conversations were frequently time-constrained, offering little time for individual concerns to be addressed or in-depth explanations by physicians. Neurologists specialising in PD were perceived as particularly supportive, but the search for them posed an initial challenge for those affected.

Relatives found it particularly burdensome that current information about available services and their availability is difficult to access. The information they and their affected family members encountered was perceived as extensive and confusing, contributing to a sense of contradiction. Medication intake was a central concern, yet knowledge gaps exist regarding its mechanisms and side effects. Navigating the healthcare system and understanding support and care structures, including assistive devices, were considered challenging and deficient. A holistic needs assessment for PwPD and their families is lacking, as is professional guidance throughout the disease process. Relatives often felt abandoned, which, together with assuming caregiving responsibilities, experiencing pressure, and the absence of specific, established resources, led to psychological distress. They clearly expressed the need for psychological support.

## Theme 2 - PD management support offers

### Subtheme 1 and 2

#### The views of PwPD regarding supporters and the type of support

PwPD recognised different support groups within the healthcare system: outpatient professionals, especially their general practitioner and neurologist, and specialised clinics. Moreover, individualised support activities or training were occasionally provided by physiotherapists, occupational therapists, psychotherapists, neurologists, speech therapists, nutrition counsellors or spiritual advisors. Moreover, they perceived that the national and regional PD self-help associations offered support (e.g. regional self-help groups). In these cases, the coordinator of the self-help group acted as a contact person for further issues (e.g. complex questions), which was highly appreciated by the PwPD. Unfortunately, these different groups seldom interact with each other.

Most PwPD did not receive any training or utilise any support services besides self-help groups. Around 25% of the PwPD participated in some form of individual therapeutic training (e.g. offered by the physiotherapist or speech therapist) or attended PD information sessions (e.g. offered by PD specialised care centres). Younger PwPD found it especially challenging to find programs, groups, or other support offerings that were suited to their needs and appropriate for those who are younger or at earlier PD stages. Only some PwPD attended topic-specific information sessions (e.g. focusing on new treatments or nutrition) offered by clinics or organised self-help groups.

#### Relatives’ views regarding supporters and the type of support

Many relatives knew of existing support services, but almost all stated they had not received any training or assistance besides self-help groups. Those whose partners had visited specialised PD clinics described them as very helpful. Neurologists and other physicians only sporadically helped connect their affected relatives to any groups or services.

Some relatives visited informational assemblies or other informative events and considered them very valuable. Moreover, the relatives considered therapies (e.g. physiotherapy, occupational therapy or speech therapy) as fundamental to their management strategies, not only regarding the effects of the specific PD-related symptoms but also from a psychosocial perspective.

## Theme 3 - self-management strategies

### Subtheme 1

#### The views of PwPDs on achieving comprehensibility

When asking for a self-assessment of the comprehensibility of their disease, the level of information provided to PwPD varied widely. The process of obtaining information differed based on the duration and severity of PD and the willingness of PwPD to engage with the available material. PwPD with higher Hoehn and Yahr stages reported less need for information. Those diagnosed relatively recently estimated their knowledge as ‘bad’ to ‘mediocre’ or ‘satisfactory’, although almost all stated that there was room for improvement. Only two participants stated they felt no need for further information. A few mentioned that they intentionally kept their knowledge low due to a fear of becoming overburdened.

When the PwPD were asked about the sources and channels of information they used, their answers were again very diverse. Almost all had regularly used the internet. Other important channels were, along with their physicians, self-help groups and PD associations, family and friends, print media, television and special events hosted by hospitals or PD clinics. Occasionally, nursing services or outpatient physiotherapists provided information.

Many of the PwPD revealed how burdensome the condition was for their relationships. They frequently talked about the sorrow they feel when seeing their family members suffer because of their PD diagnosis. Some mentioned that they were secretly hoping to die before depending too heavily on their partner’s care. The distribution of roles and the dynamics within their relationships were extremely diverse; the PwPD were the dominant force for managing their condition in some cases, while their partner took over this role in others. Some PwPD frequently perceived a lack of understanding from their partner and wished for them to be better informed about the symptoms of PD. For one PwPD, their partner terminated their relationship right after their diagnosis. However, most PwPD expressed gratitude for their relative’s support.

#### Relatives’ views on achieving comprehensibility

Throughout, all relatives had a general understanding of the basics of PD. However, most stated that significant room for improvement existed regarding their knowledge. Again, those with partners in the later stages expressed less need for information.

The intensity with which the relatives had immersed themselves in the topic differed greatly. While some had been the driving force in understanding all circumstances, others only knew what their affected partner had told them. When asked about topics for which they still felt the need for more information, their answers centred on the same subjects as in the PwPD: general information about PD, including its symptoms, causes and progression; possible treatments, therapies and medication; the future need for caregiving, support options and entitlements for healthcare and financial support.

Channels used for gathering information were primarily the internet: *‘Your only friend is Google’ (*R 22 of PwPD, H&Y stage 2–3), followed by the responsible medical professionals. The relatives further consulted their family and friends, print media, television, and, in some cases, informative events hosted by clinics, private PD support groups or official PD associations. *‘I did most of the research myself. There is not much coming from the physicians’* (R 19 of PwPD, de novo).

### Subtheme 2

#### The views of PwPD on achieving meaningfulness

After initially strong emotional reactions, most PwPD reported they had found ways to cope relatively well with PD and had accepted it. The PwPD described appreciable individual differences during this process. Many in advanced PD stages explained that working on their mindset and finding a positive attitude towards their PD was necessary.

A few attempted to only concern themselves with their illness as little as necessary and continue their familiar routines, as far as possible in a sense that the *‘PD should not dominate’* (PwPD 52, H&Y stage 1).

#### Relatives’ view on achieving meaningfulness

In some cases, the relatives accepted the new situation and took a more carefree stance towards the condition. In others, the relatives tended to ignore the disease and symptoms of the PwPD to protect themselves.

Overall, relatives attempted to offer encouragement and support to their affected relatives through active involvement in managing necessary treatments and therapy, a positive mindset, and open communication. Open communication about PD and its symptoms and progression was particularly challenging for many relationships.

### Subtheme 3

#### The views of PwPD on achieving manageability

Most of the PwPD found ways to actively shape their *‘life 2.0’* (PwPD 17, H&Y stage 3), including new hobbies, new social networks and adjusting daily routines. Some actively disclosed their condition to get along with others.

All PwPD stated that it was helpful for them to *‘not to look too far into the future’* (PwPD 4, H&Y stage 2–3) and only concern themselves with their current or upcoming PD stage at most. When asked how they manage their condition in daily life, the younger PwPD usually responded with information about their work life and children. Family support eases the burden of managing PD. However, those who still work found it challenging to open up about their diagnosis; nonetheless, when they did, it was often perceived as relieving. PwPD actively sought support in their daily lives through medical aids or household assistance.

#### Relatives’ views on achieving manageability

The relatives described diverse challenges they had faced since their affected relatives had been diagnosed and adjusted to their new lives. Many initially felt overwhelmed by the situation. Witnessing the changes in their affected relatives’ behaviour, character, and mental and cognitive well-being caused them great distress.

Many relatives also struggled with resistance and defensive attitudes toward medical treatment, physical activity or other forms of therapy on behalf of their affected relatives. Time management was a further issue, especially when balancing work and caregiving. Young families with children faced particularly difficult situations in terms of explaining the disease to their children. Most who still worked had adjusted their work conditions to accommodate their partner’s needs. However, many stated that in adjusting to the new situation, they inevitably had to prioritise their partner’s needs over their own and tended to place themselves in the background.

Some relatives talked about their search for support networks (i.e. family caregiver support groups and self-help groups) and had both positive and negative experiences. Those who found suitable groups appreciated the sense of community, the support, and the openness in discussing PD-related topics. However, some were less successful in their search for a suitable support network. Locally available self-help groups were often not perceived as appropriate for their individual needs, and in some cases, being confronted with PwPD at higher Hoehn and Yahr stages caused severe fear of progression for those whose partner had only recently received the diagnosis.

Most relatives considered physical activity and exercise programs crucial and integrated them into their daily lives with PD. The relatives also demonstrated a strong willingness to accompany their affected partners to these activities and, in some cases, participated themselves.

It became apparent that orientation within the healthcare system and support structures outside the healthcare system depended strongly on the self-initiative of the relative or their affected partner, respectively.

## Theme 4 - future needs

### Subtheme 1

#### The perceived needs of PwPD

Most of the PwPD desired a holistic and comprehensive healthcare concept and early offers of structured capacity-building activities (e.g. patient school). In such training, the PwPD wished to gain a condensed overview of PD as a whole, with a high demand for *‘valid information about nutrition*’ (PwPD 21, H&Y stage 4) and information on medication and possible side effects; the same was true for comorbid conditions, such as depression. They especially desired more orientation about existing support structures.

Many wished to be more connected to other PwPD (e.g. in self-help groups), ideally with those of a similar age and PD stage. Several participants expressed a desire for psychological support. Finally, since family members play an important role, their support is also considered ‘*obligatory*’ (PwPD 52, H&Y 1).

#### Relatives’ perceived needs

Most importantly, relatives’ desires were consistent with those of the PwPD in requesting a holistic care concept for PD. They also expressed the need for networking: Self-help groups should also focus on the partners of those affected by PD.

### Subtheme 2

#### The deficits perceived by PwPD

The greatest deficits perceived by PwPD were the availability of structured information, orientation and lack of time on the part of physicians. The PwPD felt challenged by the lack of navigation between different non-collaborating healthcare professionals and institutions. Many felt lost in the maze of possible therapies and interventions, entitled benefits and different medical professionals who only sporadically collaborated. In addition, insufficient information about the side effects of medications was expressed as very problematic. The PwPD reported deficits in the PD-specific capabilities of the medical professionals. Some described harmful experiences while staying in the hospital for something non-PD related and being treated with medication that was not previously aligned with their PD medication or not suitable for PwPD in the first place.

While the internet was an important information channel, most of the PwPD perceived the amount and quality of (sometimes discrepant) information as overwhelming and unsettling. Their information needs depended on how involved or dedicated their neurologist was and *‘what bridges they build or don’t build’* (PwPD 24, H&Y stage 2–3).

Finally, the societal perception of PD was described as *‘very difficult’* (PwPD 29, H&Y stage 2–3). Some PwPD had experienced staring or comments from others in public that were caused by a lack of understanding of PD symptoms. This distorted public image was described as burdensome and placed PwPD at risk of social isolation.

#### Relatives’ perceived deficits

The relatives perceived substantial deficits in information about available support services and their accessibility. Time constraints on the part of the responsible physicians deprived them of opportunities to ask questions and discuss available treatment options in more depth. They considered treatment management a critical issue, yet knowledge gaps persisted in how medications worked and their potential side effects. They perceived navigating the healthcare system and understanding support and supply structures as significant challenges that could have been simplified.

With their caregiving responsibilities, the relatives felt tremendous pressure, and the absence of concrete, established support offers for family members caused them distress. Societal perceptions and attitudes toward PD were perceived as further complicating caring for their affected partner.

Further illustrative quotes are provided in Table 3.

**Table 3:** Themes and corresponding quotes from PwPD and their relatives

Themes and subthemes	Examples of the perspectives of PwPD	Examples of the perspectives of therelatives of PwPD
Theme 1: Experiences		
Experiences with diagnosis	*‘I could not quite grasp the diagnosis. I needed some time for it. I got my diagnosis in December, and only now I am slowly figuring out how to deal with it.’* (PwPD 38, de novo)	*‘I used to be a nurse, and I did some Google research, and I found it pretty obvious. Looking back, it is unbelievable that it was not diagnosed earlier. I think that’s horrible.’* (R 16 of PwPD, de novo)
	*‘How many years do I have left? I immediately had those images in my head – Mohammed Ali, Ottfried Fischer… and I was asking myself: How long until I act like that?’* (PwPD 52, H&Y stage 1)	*‘Well, I did not know much about Parkinson’s before. The first questions were, of course, What kind of disease is that? What does it do to a person? Is it curable? Is it not?’* (R 9 of PwPD, H&Y stage 2–3)
Experiences with care provision	*‘Two days ago, I visited my neurologist, and she said, “Keep taking that medication, and I will also write down something else for you.” Yesterday, I went to the pharmacy, and they told me: “We cannot give you that. There is a clear incompatibility.” They told me I have to stop taking the medicine that I was prescribed two days ago. Now I do not know what is right.’* (PwPD 35, de novo)	*‘My husband got his diagnosis in 2017… I was pretty sure, but he did not want to hear anything about it. He did not ask anything!’* (R 22 of PwPD, H&Y stage 2–3)
Theme 2: Management support offers		
Supporters	*‘For me, it was just like that after the diagnosis; nothing came from the cash register, there were no offers. Neither from the family physician, yes, nor from the neurologist.’* (PwPD 20, H&Y stage 3)	*‘I would like a group where you really just bring together the relatives.’* (R 9 of PwPD, H&Y stage 2–3).
Type of support	*‘Neurologists don’t tell you about self-help groups. Maybe they mention it or give you a brochure, but that is it.’* (PwPD 44, H&Y stage 2–3)	*‘Or that the neurologist also points out that there is a self-help group and that you should train yourself. That doesn’t happen at all!’* (R 7 of PwPD, H&Y stage 5)
Theme 3: Self-management		
Comprehensibility	*‘When I first had the diagnosis, I felt so clueless. I also didn’t want to jump onto the internet because there you find so much, and that just scares you. But to know how to face Parkinson’s, I needed more information.’* (PwPD 4, de novo)	*‘I did most of the research myself. There is not much coming from the physicians’* (R 19 of PwPD, de novo).
	*‘Right now, the flow of information is organised more like a lottery’* (PwPD 52, H&Y stage 1).	*‘Today, I already would like to know how to better handle the situation and to know about when something new is discovered – who can you turn to? What is possible? Where can you ask? But in the end, you are mostly alone.’* (R 2 of PwPD, H&Y stage 1)
Meaningfulness	*‘I always call him “Little Parkinson”. He always runs next to me; he’s always with me; he might overtake me at some point. Yes, for me, that is Mr Parkinson’* (PwPD 49, de novo).	*‘You have to have a positive attitude towards it. That is crucial!’* (R2 of PwPD, de novo)
	*‘Today, I actually don’t see Parkinson’s as a disease, but rather as a mild impairment.’* (PwPD 39, H&Y stage 1)	*‘I do not let it inconvenience me. Michael J Fox has been living with it for years’* (R 12 of PwPD, H&Y stage 3).
Manageability	*‘To be honest, I don’t really want to know too much. Some time ago, I was at the airport, and I saw a man in a wheelchair. I think he had the same illness; he had a tremor and looked pretty broken. I don’t want to look too far ahead. I don’t know if this is good or bad. I just do not want to see these images.’* (PwPD 51, de novo)	*‘Parkinson’s is something that both partners have together. The partner needs to be just as well informed as the patient.’* (R 9 of PwPD, H&Y stage 2–3)
	*‘At work, my colleague said “seems like you drank too much yesterday” because I was shaking. “No”, that is my friend Parkinson.” What else can you do?’* (PwPD 7, H&Y stage 1)	*‘You can be well trained, but when you spend every day with your affected partner, you start becoming a solo fighter. So you run the risk of forgetting about yourself or being so tense that it only takes a tiny little thing – and that’s it.’* (R 12 of PwPD, H&Y stage 4–5)
Theme 4: Future needs		
Deficits	*‘I expected more from my physician. I have the feeling he is overwhelmed with the number of patients in his waiting room. He gives you a few minutes and then – next. No explanations, nothing.’* (PwPD 51, de novo)	*‘Especially those with a lower education won’t survive in the jungle. There is a great social injustice.’* (R 22 of PwPD, H&Y stage 2–3)
	*‘I was in the hospital because of a lung infection, and I said: “I have Parkinson´s disease”. The physician looked at me and said: “You can’t have Parkinson’s. You are too young”. I told them: “I cannot take certain antibiotics; some are incompatible with my medication”. I ended up in a wheelchair. I wish people were better informed.’* (PwPD 47, H&Y stage 2–3)	*‘Everybody asked how my husband was doing. Nobody asked how I was doing.’* (R 21 of PwPD, H&Y stage 2–3)
Needs	*‘When you have diabetes, they immediately receive training in a diabetes school. There are physicians who are responsible for that. They have to visit regularly. They get a check-up. Why not for Parkinson’s? Some people just get left alone.’* (PwPD 49, de novo)	*‘I wish there was psychological support for the relatives, for the family caregivers. That we get supported too!’* (R 17 of PwPD, H&Y stage 4–5)
	*‘Pensions for reduced earners, all of that stuff, the care package that we are entitled to. We didn’t know any of this, and it is not that easy to understand what you can and can’t request. That would be important to know.’* (PwPD 45, H&Y stage 2–3)	*‘There is someone, and they say to you, “Look over there, the drug addict or the drunk with four children”.’* (R 20 of PwPD, H&Y stage 2–3)

## Discussion

Our study aimed to comprehensively explore the lived experiences of German PwPDs and their relatives with PD, PD-related care provision, and how they manage PD and their needs. To our knowledge, this study is the first detailed and multicentre exploration of the lived experiences of PwPD and their relatives, including those with the PD diagnosis process and care provision, and examination of their associated needs.

### The experiences of PwPD and their relatives

The diagnosis, universally characterised by PwPD and their relatives as a *‘life-interrupting event’* (PwPD 2, H&Y stage 1), elicited sentiments akin to *‘standing in the rain without an umbrella’* (PwPD 8, H&Y stage 2–3) or being under shock and facing the fear of progression. At this critical moment, the lack of support system guidance, coupled with the deficiency or unstructured provision of valid or comprehensible information (e.g. about the side effects of medical treatment), was experienced as increasing their emotional burden, manifesting as feelings of anxiety, overburdening, repression or helplessness.

Most PwPD experienced medical support from outpatient neurologists, general practitioners or specialised clinics; unfortunately, the existence of regional self-help groups was noted only in some cases. Several PwPD across all disease stages complained that they had not been offered individual training, and only some felt that information sessions (e.g. within clinical settings) could help to *‘quench their thirst for knowledge’* (R 7 of PwPD, H&Y stage 4–5). Especially in the early PD stages, most PwPD felt abandoned and had difficulty mastering their new circumstances. However, over time, they obtained self-management strategies, leading to an enhanced understanding of PD, giving it meaning (e.g. acceptance) and facilitating the navigation of their ‘life 2.0’. However, their learning about the disease and constructing of these strategies were mainly self-directed.

Notably, our study underscored various deficiencies within the existing PD care support system. Pronounced issues included the lack of structured and adequate information provision, challenges in support system orientation and constraints related to the time and capabilities of healthcare professionals. Since many participants experienced discriminating or intimidating situations, the social perception of PD was perceived as *‘immature’* (PwPD 47, H&Y stage 2–3). Consequently, PwPD and their relatives require a holistic and comprehensive PD care concept. A structured training program that targets developing self-management strategies is needed to address these challenges efficiently. Furthermore, investing in network building, peer and relative support, and offering psychological support, especially during the *‘so-called PD honeymoon period’* (PwPD 1, H&Y stage 1), were considered obligatory.

Our findings showed that the participants’ experiences were heterogeneous. Most harmful experiences were associated with how the diagnosis was delivered and the lack of a comprehensive support system for PD care. While various studies have described the general physical and psychological challenges of living with PD [5, 7, 14], more extensive efforts are needed to explore the context-specific challenges and support needs of PwPD and their relatives, aiming to mitigate PD symptoms and address the requirements of PwPD and their relatives. Promoting the future well-being of those affected aligns with previous studies [17, 35].

In our study, many of the PwPD experienced the communication about their diagnosis and the conditions under which it was delivered as inappropriate and intimidating. They attributed this to a lack of empathy, insufficient time for questions, limited informational material, and the minimal involvement of relatives. This observation seems similar to a Dutch study, where PwPD and their relatives desired better information and emotional support from healthcare professionals and greater active involvement in clinical decision-making [17]. In order to ease this situation, we recommend that healthcare professionals invest further in refining their communication skills and developing therapeutic relationships. Following protocols that are abbreviated as SPIKES, BREAKS or ABCDE may help increase communication quality when delivering a diagnosis perceived as ‘bad news’ [36–38].

### PD management support offers

The PwPD and their relatives described the actual PD care support they received as unstructured, with their trajectory being influenced by the physicians’ deliberation and system orientation. While there are promising self-help and care structures in Germany, they are unfortunately locally organised and not universally accessible to all PwPD [20]. Ethical considerations underscore the need for advocacy and compassion for PwPD and their families [11]. Regrettably, only some PwPD are immediately connected to support structures through the initiative of their neurologists; some were informed, and others were not.

Accessibility to different types of support systems, comprehensive care across health sectors, and acknowledgement of PwPD and their relatives in managing their health are vital for managing PD [39]. Many PwPD rely on locally organised self-help groups. However, the existence and operational level of these groups decreased during the COVID-19 pandemic, and some groups no longer exist (e.g. due to the lack of group leaders or meeting rooms). Since these groups have demonstrated the potential to enhance PD adjustment, reduce psychiatric symptomatology, and increase coping skills and life satisfaction [40, 41], we recommend considering organised self-help groups as an integral component of the PD support system.

In the German healthcare system, PwPD typically have appointments with their neurologist every three months. However, many participants expressed the need for comprehensive information and system orientation along their trajectory. While a medicolegal barrier exists in Germany, distributing a list of PD-experienced therapists (e.g. speech therapists, physiotherapists or psychologists) could support the orientation needs of PwPD [42]. Moreover, the joint investment of self-help groups in designing understandable, tailored information material (e.g. by applying quality check support instruments such as the User-friendly Patient Information Material Checklist [UPIM-check]) or applying a patient-centred needs questionnaire might be advantageous for both affected families and health professionals [17, 43, 44]. Engaging with relatives as early as possible is considered helpful, particularly in preparing them for their future roles [12]. Specifically, regarding the management of multiple PD drugs, physicians could explicitly invite relatives to join their consultations with the PwPD to ensure compliance.

### Self-management

Our findings indicate that PwPD and their relatives differ in how they cope with PD, but they share many of their needs and desires, which is consistent with previous studies [35, 45]. Wieringa et al. highlighted that maintaining a coherent sense of self, feeling in control and holding a positive mind set were imperative for managing PD [35]. While obtaining self-management strategies is considered vital for managing chronic progressive diseases such as PD, our participants expressed regret that no self-management programs were available. We agree with the recent research by Tuijt et al. on PD self-management, which identified medication management, physical exercise, self-monitoring methods, psychological strategies, maintaining independence, encouraging social engagement, and providing knowledge and information to both PwPD and their relatives as crucial [45].

In Germany, patient-centred care is considered important but has not yet been reached [20]. Providing appropriate self-management strategies will empower PwPD to share decision-making and lead to a patient-centred approach [17]. We encourage offering systematic and tailored self-management as soon as possible after diagnosis to help reduce misunderstandings, health anxiety, and fear of progression and possibly empower the entire family system. Moreover, further research is needed to identify the self-management needs of PwPD and their relatives, considering factors such as demographics and the Hoehn and Yahr stages [45].

### Future needs

Our findings underscore the lack of a holistic and comprehensive PD care concept in Germany, particularly one emphasising self-management and care coordination. Similar deficiencies have been observed in other European countries. Navarta-Sanchez et al. identified four unmet needs: (i) personalised care for changing needs, (ii) accessibility of different types of support systems, (iii) comprehensive care across health sectors and (iv) acknowledgement of PwPD and their relatives in managing their health [39]. Vlaanderen et al. also noted that self-management, interdisciplinary collaboration between healthcare professionals, time to discuss the future and a single point of access to healthcare professionals are insufficiently addressed in the Netherlands [46]. The authors of both studies argued the need for a holistic, patient-centred, and collaborative care approach [39, 46]. The Dutch integrated care model seems promising when aiming to improve the quality of life of PwPD [47]. Such initiatives to create integrated PD care networks exist in some regions of Germany and have shown significant improvements in the quality of life of PwPD compared to the standard neurological practice [48].

Our data show that the current quality of care for PwPD predominantly depends on strong self-initiative and, in some cases, on coincidence. Since increased satisfaction with PD care may increase treatment compliance and outcomes, consideration should be given to involving PwPD in their care. Therefore, a holistic, comprehensive and universally accessible PD care concept is urgently needed in Germany, as our findings suggest. While we can learn from such programmes in Sweden and the Netherlands, a contextual adaption will be required [20, 47, 49, 50]. During the programme´s developing and piloting phase, the action research approach will be applied [51]. Elements such as structured and adequate information provision (e.g. PD and its treatment), system orientation, network building, peer support and improvements in social perception should be addressed. Furthermore, a central ‘point of contact’ [46] (e.g. a PD nurse) to support newly diagnosed patients and their relatives would be beneficial [52]. This service must be available to all and actively highlighted by neurologists or other responsible physicians. Valuable insights can be gained from new integrated care models such as PRIME-Parkinson (Proactive and Integrated Management and Empowerment in Parkinson’s Disease) [53].

### Strengths

With 81 participants, this is considered a substantial qualitative study [26]. By generating qualitative mixed methods data through FGIs, PIs and IIs, we gained a comprehensive understanding of the lived experiences and needs of PwPD and their relatives in Germany. Conducting FGIs was perceived as valuable by both the researchers and the participants. The flexible and intensive interaction with other participants during FGIs facilitated the identification of interlinkages (e.g. how missing information leads to underestimation of medication side effects) and a nuanced understanding of specific needs at the different PD stages (e.g. distinguishing the needs of newly diagnosed PwPD and those ay later Hoehn and Yahr stages), age specific needs or geographic differences. Using IIs, PIs, and FGIs allowed us to address participants’ wishes (e.g. including those at later Hoehn and Yahr stages or who did not feel comfortable joining a group discussion), which could be relevant beyond the German context.

The semi-structured interview guidelines and the researchers’ methodological experiences facilitated data collection, providing flexibility in exploring emerging insights. Validity was enhanced by the researchers directly addressing any misunderstandings or uncertainties. While the participant groups were not entirely equivalent among the Hoehn and Yahr stages, we gained a representative qualitative sample. Moreover, the support of the self-help group enabled us to highlight the needs of younger PwPD. We consider our sample size sufficient since saturation was reached.

### Limitations

Our data are specific to the German context, so their transferability to other settings and healthcare systems might be limited. However, the fact that data were collected in various regions across Germany, each with its distinct characteristics (e.g. infrastructure, urban/rural profiles and sociocultural influences from East to West), lends a degree of generalisability to our results, potentially making them applicable outside of Germany. In addition, only two study participants had migrant backgrounds, possibly because a good command of German was required. Future research should explore the cultural-specific needs of PwPD.

The dearth of suitable participants in some settings forced us to conduct PIs or IIs, even when applying strategies to reduce recall bias in interview studies [54]. It became evident that PwPD with cognitive impairments (H&Y 4–5 stages) found it challenging to participate in our study, with less than 10% of the participating PwPD coming from this group. A bias in willingness to participate also became apparent, especially among newly diagnosed PwPD.

We attempted to reduce the limitations related to subjectivity in the data coding and analysis by having two coders independently code all materials and then discuss them until a consensus was reached.

## Conclusions

We gained a thorough understanding of the current situation of German PwPD and their relatives (e.g. their diagnosis experiences and management support offers), self-management strategies, and further needs (e.g. comprehensive training). Our findings provide clinicians with unique insights into how PwPD and their families perceive current support, and offer practice-based suggestions for improvement. The study may raise awareness among health professionals that PwPD and their families need comprehensive care that goes beyond medical or pharmaceutical treatment. The results of the study do serve as a foundation for developing and implementing a needs-driven, comprehensive PD care support program in Germany. Such a program should address the needs of PwPD and their relatives from the initial stages of their PD trajectory. Offering such a program should help comprehensively address understanding PD, obtaining self-management strategies, building a support network and becoming an expert on one’s disease. It should address the needs of PwPD and their families from the earliest stages of their PD journey. This approach is of high clinical relevance as it has the potential to improve the quality of care and reduce the burden of illness, health anxiety and fear of progression in PwPD.

### Electronic supplementary material

Below is the link to the electronic supplementary material.

Supplementary Material 1

## Data Availability

Due to the sensitive nature of the questions asked in this study, the respondents were assured that the raw data would be kept confidential and not be shared.

## References

[1] Tolosa E, Garrido A, Scholz SW (2021). Challenges in the diagnosis of Parkinson’s disease. Lancet Neurol.

[2] Heinzel S, Berg D, Binder S (2018). Do we need to rethink the epidemiology and Healthcare utilization of Parkinson’s Disease in Germany?. Front Neurol.

[3] Zhao N, Yang Y, Zhang L (2021). Quality of life in Parkinson’s disease: a systematic review and meta-analysis of comparative studies. CNS Neurosci Ther.

[4] Rutten S, van den Heuvel OA, de Kruif AJTCM (2021). The subjective experience of living with Parkinson’s Disease: a Meta-ethnography of qualitative literature. J Parkinsons Dis.

[5] Khoo TK, Yarnall AJ, Duncan GW (2013). The spectrum of nonmotor symptoms in early Parkinson disease. Neurology.

[6] Todorova A, Jenner P, Ray Chaudhuri K (2014). Non-motor Parkinson’s: integral to motor Parkinson’s, yet often neglected. Pract Neurol.

[7] Nègre-Pagès L, Grandjean H, Lapeyre-Mestre M (2010). Anxious and depressive symptoms in Parkinson’s disease: the French cross-sectionnal DoPaMiP study. Mov Disord.

[8] Martinez-Martin P, Rodriguez-Blazquez C, Kurtis MM (2011). The impact of non-motor symptoms on health-related quality of life of patients with Parkinson’s disease. Mov Disord.

[9] Haahr A, Groos H, Sørensen D (2021). Striving for normality’ when coping with Parkinson’s disease in everyday life: a metasynthesis. Int J Nurs Stud.

[10] Geerlings AD, Kapelle WM, Sederel CJ (2023). Caregiver burden in Parkinson’s disease: a mixed-methods study. BMC Med.

[11] Bhimani R (2014). Understanding the Burden on caregivers of people with Parkinson’s: a scoping review of the literature. Rehabil Res Pract.

[12] Mosley PE, Moodie R, Dissanayaka N (2017). Caregiver Burden in Parkinson Disease: a critical review of recent literature. J Geriatr Psychiatry Neurol.

[13] Lau K-M, Au A (2011). Correlates of Informal Caregiver Distress in Parkinson’s Disease: a Meta-analysis. Clin Gerontologist.

[14] Morel T, Cleanthous S, Andrejack J (2022). Patient experience in early-stage Parkinson’s Disease: using a mixed methods analysis to identify which concepts are Cardinal for Clinical Trial Outcome Assessment. Neurol Ther.

[15] Churm D, Dickinson C, Robinson L (2022). Understanding how people with Parkinson’s disease and their relatives approach advance care planning. Eur Geriatr Med.

[16] Lidstone SC, Bayley M, Lang AE (2020). The evidence for multidisciplinary care in Parkinson’s disease. Expert Rev Neurother.

[17] van der Eijk M, Faber MJ, Al Shamma S (2011). Moving towards patient-centered healthcare for patients with Parkinson’s disease. Parkinsonism Relat Disord.

[18] Grosset KA, Grosset DG (2005). Patient-perceived involvement and satisfaction in Parkinson’s disease: effect on therapy decisions and quality of life. Mov Disord.

[19] Lorig KR, Holman H (2003). Self-management education: history, definition, outcomes, and mechanisms. Ann Behav Med.

[20] van Munster M, Tönges L, Loewenbrück KF (2020). Building a Parkinson-Network-experiences from Germany. J Clin Med.

[21] Eggers C, Wellach I, Groppa S (2021). Versorgung Von Parkinson-Patienten in Deutschland: Status quo und Perspektiven Im Spiegel Des Digitalen Wandels: care of patients with Parkinson’s disease in Germany: status quo and perspectives as reflected in the digital transition. Nervenarzt.

[22] Eggers C, Wolz M, Warnecke T (2020). Parkinson-Netzwerke in Deutschland: Zukunft Oder Utopie? Parkinson Networks in Germany: future or Utopia?. Fortschr Neurol Psychiatr.

[23] Timpel P, Tesch F, Müller G (2022). Versorgungssituation Von Parkinson-Patienten in Sachsen Eine sekundärdatenbasierte Analyse Der Inanspruchnahme Im Beobachtungszeitraum 2011 Bis 2019: treatment practice of patients with Parkinson’s disease in Saxony A secondary data-based analysis of utilization in the observation period 2011–2019. Nervenarzt.

[24] Kessler D, Liddy C (2017). Self-management support programs for persons with Parkinson’s disease: an integrative review. Patient Educ Couns.

[25] Craig P, Dieppe P, Macintyre S (2008). Developing and evaluating complex interventions: the new Medical Research Council guidance. BMJ.

[26] Morse JM, Cheek J (2014). Making room for qualitatively-driven mixed-method research. Qual Health Res.

[27] Greene JC (2007). Mixed methods in social inquiry.

[28] Robinson OC (2014). Sampling in interview-based qualitative research: a theoretical and practical guide. Qualitative Res Psychol.

[29] Vasileiou K, Barnett J, Thorpe S (2018). Characterising and justifying sample size sufficiency in interview-based studies: systematic analysis of qualitative health research over a 15-year period. BMC Med Res Methodol.

[30] Hoehn MM, Yahr MD, Yahr MD. *Neurology*, 1967:427–42. 10.1212/wnl.17.5.427 6067254

[31] Tennigkeit J, Feige T, Haak M, et al. Structured Care and Self-Management Education for Persons with Parkinson’s Disease: Why the First Does Not Go without the Second-Systematic Review, Experiences and Implementation Concepts from Sweden and Germany. J Clin Med. 2020;9(9). 10.3390/jcm9092787. [published Online First: 28 August 2020]. 10.3390/jcm9092787 PMC7563525 32872258

[32] Tong A, Sainsbury P, Craig J (2007). Consolidated criteria for reporting qualitative research (COREQ): a 32-item checklist for interviews and focus groups. Int J Qual Health Care.

[33] Kuckartz U, Rädicker S. Qualitative inhaltsanalyse. Methoden, Praxis, Computerunterstützung: Grundlagentexte Methoden: Kapitel 6.1. Qualitative content analysis. Methods, practice, computer support: comment. Wiesbaden: Springer VS.

[34] Dresing TPT. Praxisbuch Interview, Transkription & Analyse: Anleitungen Und Regelsysteme für qualitativ Forschende. Practice Book Interview, transcription & analysis: instructions and Rule systems for qualitative researchers. 8th ed. Eigen; 2018.

[35] Wieringa G, Dale M, Eccles FJR (2022). Adjusting to living with Parkinson’s disease; a meta-ethnography of qualitative research. Disabil Rehabil.

[36] Baile WF, Buckman R, Lenzi R (2000). SPIKES-A six-step protocol for delivering bad news: application to the patient with cancer. Oncologist.

[37] Narayanan V, Bista B, Koshy C (2010). BREAKS’ protocol for breaking Bad News. Indian J Palliat Care.

[38] Rabow MW, McPhee SJ (1999). Beyond breaking bad news: how to help patients who suffer. West J Med.

[39] Navarta-Sánchez MV, Palmar-Santos A, Pedraz-Marcos A (2023). Perspectives of people with Parkinson’s disease and family carers about disease management in community settings: a cross-country qualitative study. J Clin Nurs.

[40] Charlton GS, Barrow CJ (2002). Coping and self-help group membership in Parkinson’s disease: an exploratory qualitative study. Health Soc Care Community.

[41] Markó-Kucsera M, Kullmann L, Paulik E (2018). Measuring quality of life in individuals with Parkinson’s disease attending a self-help club: cross-sectional study in Hungary. Int J Rehabil Res.

[42] Warnecke T, Lummer C, Rey JW (2023). Parkinson-Krankheit. Innere Medizin.

[43] Krieger T, Salm S, Mollenhauer J et al. User-friendly Patient Information Material Checklist - UPIM-Check 2020. 10.3390/ijerph18168773 PMC8393725 34444518

[44] van der Eijk M, Faber MJ, Ummels I (2012). Patient-centeredness in PD care: development and validation of a patient experience questionnaire. Parkinsonism Relat Disord.

[45] Tuijt R, Tan A, Armstrong M (2020). Self-management components as experienced by people with Parkinson’s Disease and their carers: a systematic review and synthesis of the qualitative literature. Parkinsons Dis.

[46] Vlaanderen FP, Rompen L, Munneke M (2019). The Voice of the Parkinson customer. J Parkinsons Dis.

[47] Bloem BR, Henderson EJ, Dorsey ER (2020). Integrated and patient-centred management of Parkinson’s disease: a network model for reshaping chronic neurological care. Lancet Neurol.

[48] Eggers C, Dano R, Schill J (2018). Patient-centered integrated healthcare improves quality of life in Parkinson’s disease patients: a randomized controlled trial. J Neurol.

[49] Macht M, Gerlich C, Ellgring H (2007). Patient education in Parkinson’s disease: formative evaluation of a standardized programme in seven European countries. Patient Educ Couns.

[50] Lang C, Timpel P, Müller G (2023). Exploration Potenzieller Barrieren für die Akzeptanz eines interdisziplinären sektorenübergreifenden versorgungsnetzwerkes für Patient*innen mit Morbus Parkinson: exploration of potential barriers for the acceptance of an interdisciplinary cross-sectoral care network for patients with Parkinson’s disease. Präv Gesundheitsf.

[51] Harorani M, Jadidi A, Zand S (2022). Spiritual care in hospitalized patients in Iran: an Action Research Study. J Relig Health.

[52] Mai T (2018). Stand und Entwicklung Der Rolle als Parkinson Nurse in Deutschland – Eine Online-Befragung: Status and development of the role as Parkinson nurse in Germany – an online survey. Pflege.

[53] Tenison E, Smink A, Redwood S (2020). Proactive and Integrated Management and Empowerment in Parkinson’s Disease: Designing a New Model of Care. Parkinsons Dis.

[54] Bergelson I, Tracy C, Takacs E (2022). Best practices for reducing Bias in the interview process. Curr Urol Rep.

