# Supplementary material for: Exploring the lived experiences of individuals with Parkinson’s disease and their relatives: insights into care provision experiences, disease management support, self-management strategies, and future needs in Germany (qualitative study)
Source: BMC Neurol. 2024 Jun 18;24:208. doi: 10.1186/s12883-024-03696-y (PMC11184701; doi:10.1186/s12883-024-03696-y)
Supplement: Supplementary file 1 — Supplementary Material 1 [file 12883_2024_3696_MOESM1_ESM.docx]

*Annex 1: Example of the topics of the semi-structured interview guideline (Part 1, status-quo assessment, perspective of the people with Parkinson disease).*

| Top question |
| --- |
| As a person living with Parkinson’s disease, how do you experience your disease and current support? |
| Ice Breaker |
| When you were diagnosed, do you remember roughly which questions mainly concerned you at the time? |
| Sub Question 1 |
| How would you consider your current level of information about Parkinson’s disease?   - Level of information - Need for information - Knowledge transfer - Information channels - Deficits |
| Sub Question 2 |
| Which training and support programmes have you used so far because of your illness?   - Support offer analysis - Knowledge of services - Experience - Support gaps |
| Sub Question 3 |
| What support should be given to people with Parkinson’s disease and their families?   - Individual level (patients or relatives) - Organisational level (e.g. within the inpatient or outpatient setting) - System level (e.g. by support organisations or health insurance) |
